# Integrated sensitive on-chip ion field effect transistors based on wrinkled InGaAs nanomembranes

**DOI:** 10.1186/1556-276X-6-215

**Published:** 2011-03-14

**Authors:** Stefan M Harazim, Ping Feng, Samuel Sanchez, Christoph Deneke, Yongfeng Mei, Oliver G Schmidt

**Affiliations:** 1 Institute for Integrative Nanosciences, IFW Dresden, Helmholtzstrasse 20, 01069 Dresden, Germany

## Abstract

Self-organized wrinkling of pre-strained nanomembranes into nanochannels is used to fabricate a fully integrated nanofluidic device for the development of ion field effect transistors (IFETs). Constrained by the structure and shape of the membrane, the deterministic wrinkling process leads to a versatile variation of channel types such as straight two-way channels, three-way branched channels, or even four-way intersection channels. The fabrication of straight channels is well controllable and offers the opportunity to integrate multiple IFET devices into a single chip. Thus, several IFETs are fabricated on a single chip using a III-V semiconductor substrate to control the ion separation and to measure the ion current of a diluted potassium chloride electrolyte solution.

## Background

The integration of ion field effect transistors (IFETs) into solid state micro-/nanofluidic systems for accurate transport control of charged species such as ions, proteins, or DNA in "Lab on a Chip" systems (LoC) is highly important in life sciences [1-5]. Recently, there is an increasing interest in IFETs due to the high demand in micro total analysis system (μ-TAS) devices dealing with bio-health, bio-sensing, and bio-physical applications [3,6,7]. Those μ-TAS devices should automate the entire analytical process, from sample processing and preparation to sensing and analysis within a small, cheap, and easy to handle system. To realize structures for simultaneous sample treatment on a single chip, it is crucial to combine (1) a semiconductor material as substrate material for microelectronic components and (2) a fabrication technique which ensures the integration and alignment of the nanofluidic channels within the chip [8].

Over the last few years, different production techniques have been developed to prepare nanochannels for ion control such as nanotubes [9-12], porous membranes [4] or ion channels [13] to name a few. The permeability of nanofluidic channels for ions can be modified by the channel size, surface charge distribution within the channel, and external electrical fields [2,5]. For extrinsic ion current control within the nanochannel, a field effect transistor electrode structure design has to be integrated [1,5,14]. New technological methods are necessary to achieve the challenging goal of fabricating accurately aligned single nanochannels, including the microelectronic integration into a μ-TAS device. Other fabrication techniques have not succeeded in the combination of all of these μ-TAS requirements. Furthermore, the fabrication of nanochannels with only one dimension in the nanometer range does not provide easy single molecule treatment. The random alignment to the substrate of some nanochannel fabrication methods leads to difficulties in the reproducibility of IFET production. As an alternative solution or to overcome these limitations, self-deterministic wrinkling in the nano regime on semiconductor materials were investigated recently and successfully tested for their fluidic capabilities. Due to the high integration state and their good alignment to the substrate, it was predicted that they could eventually be potential candidates in new lab on a chip applications [15]. Herein, we present a new method of manufacturing nanofluidic channels for IFET devices on a semiconductor material by combining our recently developed technique called "release and bond back of layers" (REBOLA) with microfluidic fabrication techniques [8,15,16]. The original REBOLA process relied on wrinkling large strained nanometer thick membranes onto semiconductor substrate materials, which allowed us to fabricate complex networks of nanochannels. We improved the REBOLA process to fabricate aligned single nanochannels of different types, instead of producing networks, to be utilized in IFET devices. Consequently, the nanochannels are integrated into a microfluidic device unit which includes the photolithographically defined electrodes for ion detection as well as the microchannel system for liquid transport. A self-limiting atomic layer deposition (ALD) of Al_2_O_3 _isolates the electrodes electrically from the substrate and fine-tunes the inner diameter of the nanochannel to match the channel height with that of the Debye length of the liquid. The Debye length expresses the thickness of the electrical double layer at the solid-liquid interface, where the electrical neutrality of an electrolyte is broken and is equal to approximately 30 nm for a 10^-4 ^M ion solution [17]. The integration of wrinkled nanomembranes into a LoC system is presented. Furthermore, the nanochannels are suitable for ion current manipulation and IFET applications, which was tested with a 10^-4^-M KCl solution as electrolyte with differing electrode parameters (from +1 V to -1 V).

## Results

### Device fabrication

The device structure consists of three assembling parts: (1) the nanofluidic channel fabrication, (2) integration of electrical components, and (3) microfluidic channel fabrication for the liquid reservoirs on either side of the nanochannel, including a top poly-dimethylsiloxane (PDMS) sealing layer.

**Figure 1:**
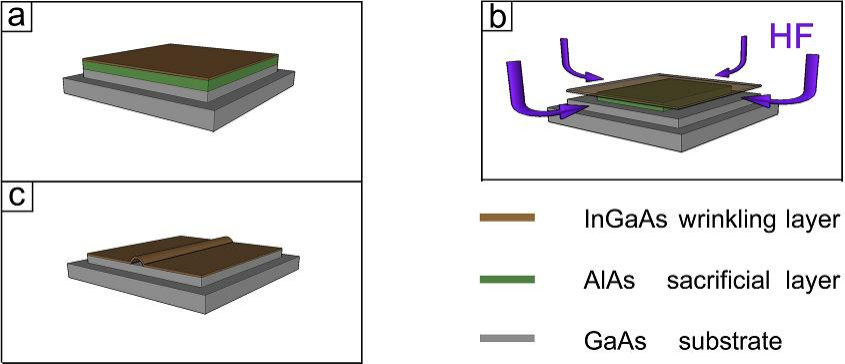
**Sketch of the general wrinkling process in three steps**. **(a) **Pre-defined wrinkling structure by photolithography and wet chemical processing, **(b) **selective HF etching of the sacrificial AlAs layer and starting release of the strained InGaAs layer, **(c) **bond back and wrinkling of the released functional InGaAs layer.

The basic substrate for wrinkling consists of a stack of three layers which was grown by molecular beam epitaxy (MBE) onto the GaAs substrate. First, a 200-nm thick GaAs film was grown as a buffer to provide a smooth and crystalline surface for the second 80-nm thick AlAs sacrificial layer. Finally, a 20-nm thick In_0.2_Ga_0.8_As functional layer was grown on top of the sacrificial layer. The fabricated stack of layers was processed into a square-shaped mesa structure with a lateral side length of 6 μm by photolithography and wet chemical etching as shown in Figure 1a. A diluted HF etchant selectively removes the AlAs sacrificial layer and released the InGaAs nanomembrane from the GaAs buffer (Figure 1b). Afterwards, due to the internal stress of the wrinkling layer, the InGaAs membrane began to bond back to the substrate and wrinkled into a straight channel-like structure (Figure 1c) [16,18].

**Figure 2:**
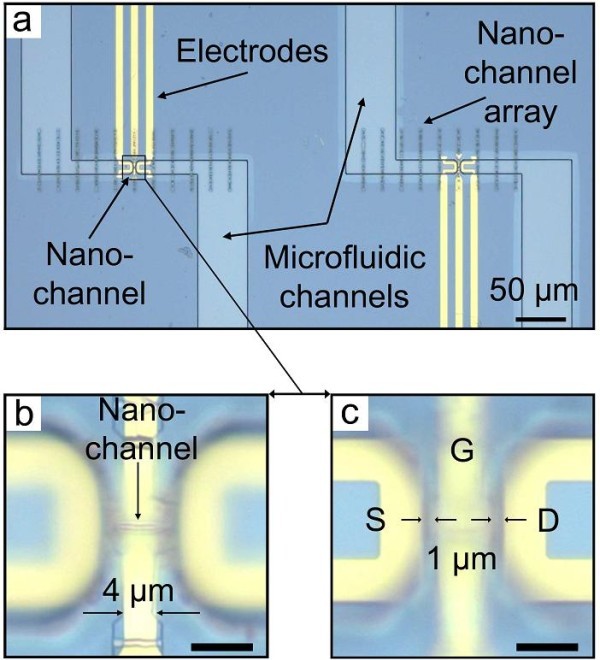
**Top view on two IFET devices**. **(a) **Optical microscope images of two devices containing one wrinkled nanochannel each, the electrodes and the microfluidic channels. **(b) **and **(c) **are the zoomed images on one device in two different *Z*-positions focusing on the nanochannel (b) and on the electrodes (c). The unlabeled scale bars are 5 μm.

After the nanochannel fabrication, the electrical isolation of the entire substrate, including the nanochannel, was carried out by an ALD method by depositing a 15-nm thick Al_2_O_3 _layer. The electrodes were then deposited in separate steps starting with the gate electrode on top of the channel. After the gate electrode integration by photolithography processing and electron beam evaporation of 80 nm of gold, a second ALD step isolated the gate electrode completely. The purpose of the second Al_2_O_3 _layer is not only to ensure the electrical isolation but also to tune the final inner diameter of the nanochannel. The ALD method allows for a homogenous coating of nanochannels with lengths in the micrometer regime [19]. This coating is of significant importance since ions with a given concentration have a defined Debye length, which needs to be in the same order as the nanochannel dimension [20,21]. The original height of the nanochannel after wrinkling, measured by AFM, was 95 nm. After the first ALD deposition of 15 nm of Al_2_O_3_, the inner height had been diminished to 65 nm. By the coating of a 20-nm Al_2_O_3 _layer from the second isolation step, the channel height finally reached a total height of 25 nm. After the gate electrode was isolated and the channel size fine-tuned to the desired value, the deposition of the source and drain electrode was also performed by a photolithography and electron beam evaporation step. The source and drain electrodes were separated by 1 μm to that of the nanochannel openings, whereas the gate electrode partially covered the top of the nanochannel (66% of the length). Figure 2 shows optical images of two IFET devices including the electrodes and the microfluidic system. Each nanochannel array shown in Figure 2a provides a large number of wrinkled InGaAs membranes (165 wrinkled membranes per array) which provide various choices of nanochannels to be selected for the IFET fabrication. Almost similar nanochannels of each array were selected to prepare the IFET devices. Figure 2a depicts two arrays of nanochannels containing the processed electrodes and microfluidic structures.

**Figure 3:**
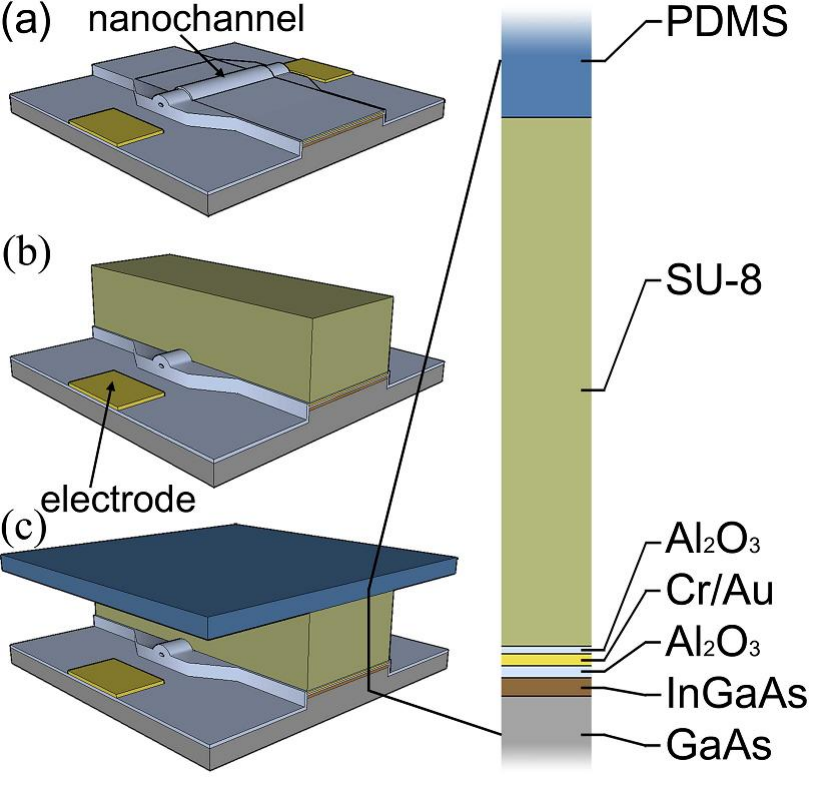
**Schematic of the microfluidic setup**. **(a) **A processed transistor structure, including the electrodes and the wrinkled nanochannel. **(b) **SU-8 microchannel walls defining the microfluidic channels on the chip structure. **(c) **Sealing of the microchannel system by using a flexible PDMS top layer.

The microchannels were defined by photolithography of SU-8 10 photoresist, which is mechanically stable and biocompatible [22-24]. By using this high viscous, negative photoresist and optimized spin-coating parameters, the microfluidic channels reach a height of 10 μm. To seal the entire channel system, a PDMS layer was prepared and placed on top of the microchannel system (see Figure 3). This top layer avoids rapid liquid evaporation and contains also connectors to attach an external micropump system to the device [25,26].

### Versatility of wrinkling

**Figure 4:**
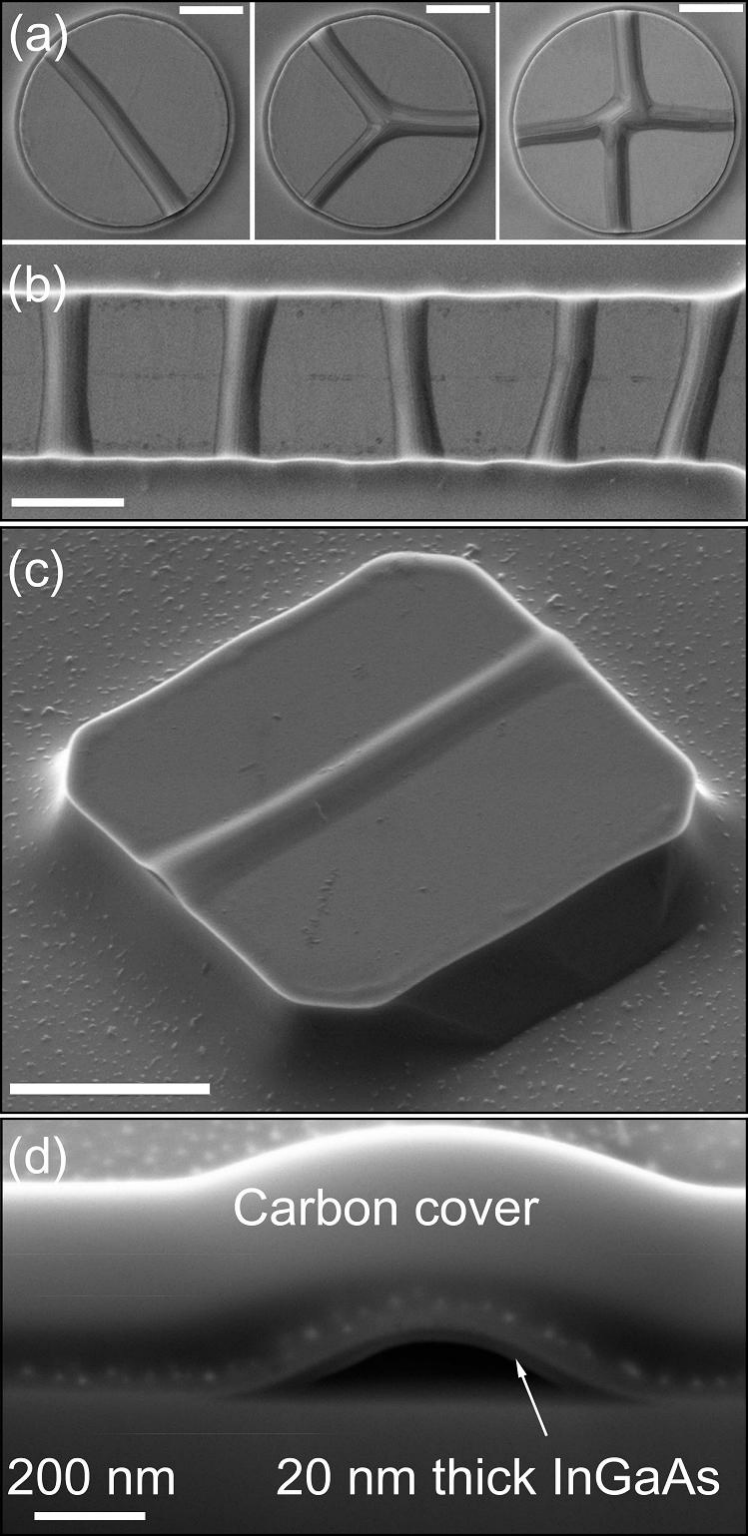
**Versatility of wrinkling**. Alternative wrinkling structures leading to different channel arrangements such as **(a) **multiple openings for circular mesa structures and **(b) **parallel alignment of channels for stripe-like mesa structures. **(c) **An SEM image of a squared GaAs mesa structure with a wrinkled In_0.2_Ga_0.8_As layer on top. **(d) **An SEM/FIB cut image of one wrinkled nanochannel. The carbon cover protects the wrinkled structure during the FIB cut. Unlabeled scale bars are 2 μm.

The improved REBOLA technique uses small strained InGaAs membranes on mesa structures which allows for the exact on-chip placement of the nanochannels. In order to have an accurate and reproducible channel alignment and to identify the most suitable wrinkling behavior for the fabrication of straight nanochannels, the wrinkling of different InGaAs membrane shapes were investigated. We aimed for the fabrication of square, circular, and stripe-shaped membranes as shown in Figure 4. Mesa structures with circular or stripe shapes lead to straight channel structures, but they also offer a bigger variety of channel types with different alignments. For an example, a circular shape with a diameter of 6 μm gives a more random distribution of the channel orientations and even different types channels themselves (Figure 4a). The most observed channel types are straight channels; nonetheless, three-way channels as well as four-way channels appear in a numerous quantity. For stripe-like mesa structures with a width of about 6 μm and a length of several tens of micrometers, parallel channels can be realized (Figure 4b). Only square-shaped InGaAs layers wrinkle almost in the same orientation every time and, therefore, this kind of structure has been used as the base pattern for the device assembling (Figure 4c). This preferred wrinkle orientation relies on the crystal structure of the sacrificial AlAs layer and the different HF etching rates along the crystal axis [8,18]. Due to the MBE layer growth, the AlAs crystal structure is similar to the substrate which is GaAs(001). The etching rates are highest perpendicular to the <110> direction of the crystal. Assuming that the edges of the functional InGaAs membrane are aligned to the <110> direction of the substrate, the two faster under-etched edges will bond back to the substrate prior to the two edges with a lower etching rate. This etching rate preference, fixes the wrinkle orientation of any square-shaped nanomembrane parallel to the <110> direction. A focused ion beam (FIB) cut of a straight two-way nanochannel is shown in Figure 4d, where the internal structure of the InGaAs wrinkles can be observed. The benefits of the self-organized wrinkling of strained InGaAs nanomembranes into nanofluidic channels are the integration into a semiconductor substrate with accurate control of location, channel type, and the orientation on the chip.

### Measurement preparation

The sample was mounted onto a chip carrier which was plugged into the measurement stage. The measurement itself was controlled by a PC software and was operated by the semiconductor parameter analyzer 4156C (Agilent, Santa Clara, CA, USA). All measurements have been done at room temperature. Prior to the ion current measurement, the system was successfully tested to have no electrical or liquid leakage, which was carried out without liquid in the system to ensure electrical isolation between each electrode and between the electrodes and the substrate. Al_2_O_3 _is stable using the current measurement conditions with a maximum electrical field strength of about 2 V/μm. The breakdown voltage for similar systems was investigated recently and is more than one order of magnitude higher [27]. The liquid leakage tests have been investigated with pure DI water and the same KCl solution as for the ion current measurement. The conductivity was always in the expected range so that no leakage was present [10].

## Discussion

**Figure 5:**
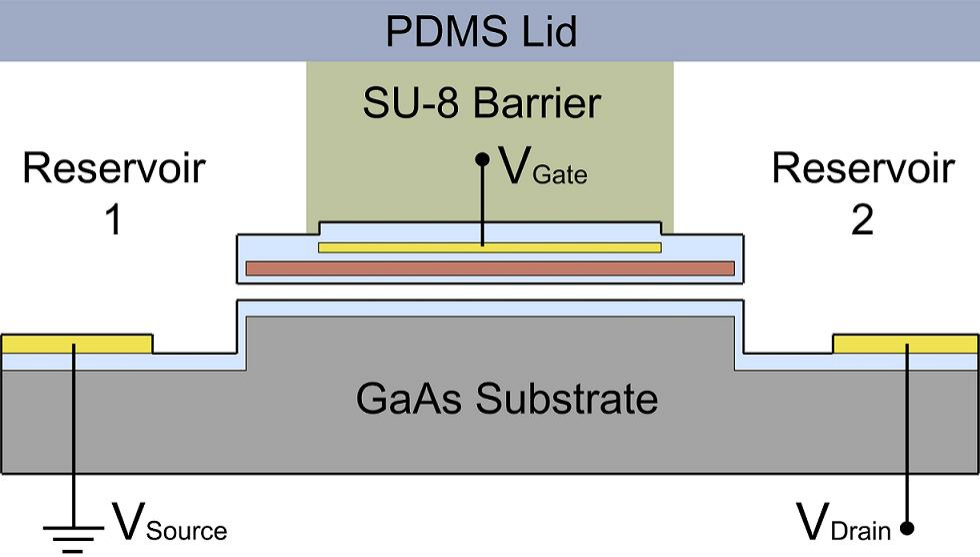
**Schematic diagram representing a cross section of one transistor device**. The two reservoirs are mainly separated by the SU-8 photoresist barrier and are only connected by the nanochannel. The top PDMS lid seals the entire system and avoids rapid liquid evaporation. The electrodes are electrically isolated by an Al_2_O_3 _layer (bright blue).

**Figure 6:**
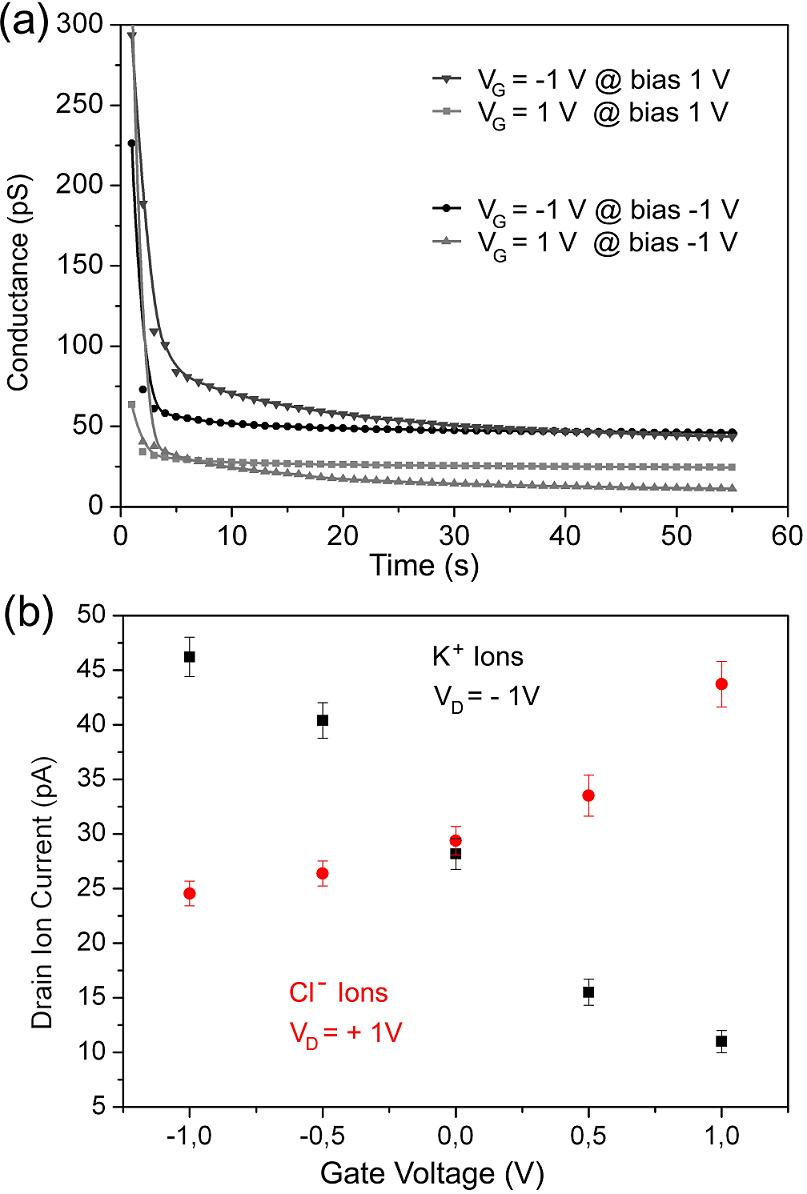
**Electrical measurements**. **(a) **Transistor conductance over time at different electrode configurations. Biases were applied at 0 s to the electrodes and the measurement started after 2 s. The fast decay of the polarization effects enables the measurement of the real current without additional effects after 55 s. **(b) **Ion current modulation by changing the gate bias while keeping *V*_S _= 0 V and setting *V*_D _= +1 V for detecting Cl^- ^ions (red points) and *V*_D _= -1 V for detecting K^+ ^ions (black points).

The current chip structure includes three independent operating IFET devices. To prove the suitability of the presented IFET devices, a 10^-4^-M KCl solution had been added to reservoir 1 and 2 of each single device (see Figure 5). The source electrode was always grounded to *V*_S _= 0 V, whereas the drain electrode had a bias of either *V*_D _= +1 V (to attract the negative chloride ions) or *V*_D _= -1 V (to attract the positive potassium ions). The measurement of the ion current is a steady state measurement, meaning that no polarization effects disturb the detection of the ion current. In Figure 6a, four conductance curves are displayed to demonstrate the fast relaxation capabilities of the presented device. For *V*_G _= -1 V and *V*_D _= -1 V the conductance decayed fast below 5% of the initial value in the first 5 s. The measurement is displayed from 3 s on, whereas at 0 s the bias was already applied to the electrodes. The data point after 55 s of each curve represents the channel conductance without polarization effects. All ion current measurements were taken in the steady state after 55 s.

The ion current manipulation had been driven in a sweep mode starting with *V*_G _= -1 V to *V*_G _= +1 V in 0.5-V steps. The ion permeability of the nanochannel for a specifically charged species can be tuned by the gate electrode while keeping all other electrode parameters constant. The measurement took place always at the drain electrode. Figure 6b shows the ion current modulation of Cl^- ^(red circular data points) and K^+ ^(black square data points) ions with changing of the gate potential. In case that there is no gate effect (*V*_G _= 0 V), the same ion current is expected, independently to the chosen source and drain parameters, since potassium and chloride ions have almost the same mobility in a liquid (76.2·10^-7 ^m^2^/sV for K^+ ^and 79.1·10^-7 ^m^2^/sV for Cl^-^) [20]. Figure 6b shows that at *V*_G _= 0 V, the ion current for Cl^- ^and K^+ ^is in the same order at about 28 pA. By increasing the gate potential to *V*_G _= +1 V, positive ions will be repelled and negative ions will be attracted to move through the nanochannel. This gate potential modification results in a lower K^+ ^and an increased Cl^- ^ion current. A mirrored behavior can be observed when the gate potential is decreased to *V*_G _= -1 V. The weak effect on the chloride ion current modulation at lower gate potentials can be explained by the permeability of PDMS for water molecules during the measurement. During this time, water molecules will diffuse into the PDMS, which can slightly increase the ion concentration in the reservoirs. Therefore, the Debye length decreases, and the gate potential loses the efficiency for ion current manipulation.

The presented IFET devices have a comparatively fast ion current detection capability in the lower pA regime [10]. The feasibility of accurate nanochannel alignment on semiconductor substrates, and the high integration state of all components are strong criteria for future continued investigations on wrinkled nanomembranes for more complex IFET systems.

## Conclusions

The fast and easy fabrication of nanochannels by the combination of the versatile REBOLA technique and standard photolithography leads to semiconductor films which form nano-sized wrinkles with useful fluidic capabilities. Different nanomembrane shapes have been prepared to investigate the most suitable wrinkling structure for IFET devices. Rectangular-shaped membranes wrinkle into straight two-way channels with a fixed orientation relative to the crystal structure of the substrate. Indeed, a circular-shaped membrane creates a higher variation of channel types, which can be used to fabricate more complex fluidic circuit, on-chip structures, but the square-shaped structures wrinkle always the same, which is more useful for reproducible IFET assembling. To demonstrate the feasibility of the integrated nanochannels for IFET on-chip devices, samples with several IFETs have been fabricated and were successfully tested for ion separation using KCl as a model electrolyte solution. The usage of a semiconductor material as a substrate and the highly integrated state of all components, including accurate channel positioning and definable channel orientation, might be highly demanded for the next integration level of "Lab on a Chip" devices. The near future approach is to adapt the system for single molecule detection, which should find a huge number of applications in bio-analytic μ-TASs. Later on, light can be used for controlling the wrinkling behavior of the nanomembrane in order to obtain more complex and deterministic wrinkle structures [28].

## Methods

### Film growth, structure reproducibility

The III-V semiconductor layers were epitaxially grown on GaAs(001) substrates. After the growth of a 200-nm GaAs buffer, 80-nm AlAs and 20-nm In_0.2_Ga_0.8_As were grown. Several samples were lithographically patterned to ensure the reproducibility of nanochannel formation. The sample dimension is always 7 by 7 mm in lateral dimension. A sample contains 12 of the so-called nanochannel arrays with 165 wrinkling structures each (see also Figure 3a). This is to increase the chance to have the same-shaped nanochannel with the same fluidic capabilities on every new sample. For reproducibility, all samples are cut along the <110> GaAs direction and the photolithographic pattern has been always aligned to the substrate structure in the same way.

### Wet etching risks and safety

The HF etchant is highly toxic. Special safety clothes are strongly recommended. The local waste disposing procedure is to be obeyed.

### Electrical measurement device and conditions

The semiconductor parameter analyzer Agilent 4156C has been used under standard environment conditions, such as room temperature and normal pressure. The measurement device has been driven in the "sweep measurement mode." Light impact on the sample as well as acoustic vibration has to be avoided at all times because of its semiconductor behavior and the high electrical sensitivity of the measurement device.

## Competing interests

The authors declare that they have no competing interests.

## Authors' contributions

SMH carried out the wrinkling studies, device assembling, electrical measurements and wrote the manuscript with contribution from SS and OGS. PF participated during the electrical measurements. CD did the MBE growth of the substrates. OGS and YF initiated the project and provided the original idea.
